# Oxidative Stress and Male Infertility: Evidence From a Research Perspective

**DOI:** 10.3389/frph.2022.822257

**Published:** 2022-02-22

**Authors:** Bashir Ayad, Temidayo S. Omolaoye, Nicola Louw, Yashthi Ramsunder, Bongekile T. Skosana, Peter I. Oyeipo, Stefan S. Du Plessis

**Affiliations:** ^1^Department of Physiology, Faculty of Medicine, Misurata University, Misrata, Libya; ^2^Division of Medical Physiology, Faculty of Medicine and Health Sciences, Stellenbosch University, Tygerberg, South Africa; ^3^Department of Basic Medical Sciences, Mohammed Bin Rashid University of Medicine and Health Sciences, Dubai, United Arab Emirates; ^4^Department of Physiology, College of Health Sciences, Osun State University, Osogbo, Nigeria

**Keywords:** oxidative stress, male infertility, semen analysis, obesity, hypertension, diabetes mellitus

## Abstract

Male fertility potential can be influenced by a variety of conditions that frequently coincide. Spermatozoa are particularly susceptible to oxidative damage due to their limited antioxidant capacity and cell membrane rich in polyunsaturated fatty acids (PUFAs). The role of oxidative stress (OS) in the etiology of male infertility has been the primary focus of our Stellenbosch University Reproductive Research Group (SURRG) over the last 10 years. This review aims to provide a novel insight into the impact of OS on spermatozoa and male reproductive function by reviewing the OS-related findings from a wide variety of studies conducted in our laboratory, along with those emerging from other investigators. We will provide a concise overview of the production of reactive oxygen species (ROS) and the development of OS in the male reproductive tract along with the physiological and pathological effects thereof on male reproductive functions. Recent advances in methods and techniques used for the assessment of OS will also be highlighted. We will furthermore consider the current evidence regarding the association between OS and ejaculatory abstinence period, as well as the potential mechanisms involved in the pathophysiology of various systemic diseases such as obesity, insulin resistance, hypertension, and certain mental health disorders which have been shown to cause OS induced male infertility. Finally, special emphasis will be placed on the potential for transferring and incorporating research findings emanating from different experimental studies into clinical practice.

## Introduction

Infertility is one of the most complicated disorders of the reproductive system. It is characterized by the inability to conceive after 1 year or longer of regular unprotected sexual intercourse (1). Worldwide, about 8–12 % of couples of reproductive age have been reported to be affected by infertility, with a widely divergent estimates of prevalence across regions and countries (2). Usually, when the attributable causes of female infertility have been eliminated and/or semen analysis results fail to meet the World Health Organization (WHO) criteria (3), male factor infertility is taken into consideration as the potential etiological factor. Males are considered to be the sole cause of infertility in almost 20% of all cases and are at least partly involved in another 30–40% (4). Despite remarkable advances in the diagnosis and management of male infertility, almost one half of all cases still idiopathic, for which no evident etiological factor has been identified (5).

In recent years, an emerging concern has been raised regarding the overall increase in male infertility rates, reflecting a global time-related deterioration in semen quality with concomitant increase in the incidence of male reproductive abnormalities (6–9). Although the particular reason for the increased incidence of male infertility remains elusive, various environmental, nutritional and socioeconomic factors have been suggested to contribute to the downward trend in semen quality (10, 11). Furthermore, common complications including obesity, dyslipidaemia, hypertension, insulin resistance as well as psychological stress and anxiety have also been associated with impaired fertility in males of reproductive potential (12–14). The link between these comorbidities and male infertility appears to be complicated and poorly understood. However, these is research-based evidence demonstrating that oxidative damage is one of the fundamental mechanisms involved in the etiopathogenesis of these illnesses (15–20). Concurrently, the critical role of oxidative stress (OS) in the development of male reproductive dysfunction has continued to gain a great deal of attention (21–24).

Redox equilibrium is essential for maintaining various vital aspects of sperm functionality. However, an imbalance in the generation and elimination of reactive oxygen species (ROS) negatively affects sperm quality due to oxidative damage (25). Owing to their imperfect antioxidant capacity and cell membrane rich in polyunsaturated fatty acids (PUFAs), spermatozoa are especially vulnerable to oxidative destruction. Under certain pathological conditions, ROS can be converted into highly reactive agents, causing dysregulation of various cellular signaling pathways and extensive damage to multiple biomolecules including nucleic acids, proteins, and lipids. The subsequent series of adverse events includes loss of membrane integrity, mitochondrial dysfunction, impaired sperm motility as well as DNA damage and apoptosis (23, 26).

The role of OS in the etiology of male infertility has been the primary focus of our Stellenbosch University Reproductive Research Group (SURRG) over the last 10 years. This review aims to provide a novel insight into the impact of OS on spermatozoa and male reproductive function by reviewing the OS-related findings from a wide variety of studies conducted in our laboratory, along with those emerging from other investigators. We will provide a concise overview of the production of ROS and the development of OS in the male reproductive tract along with the physiological and pathological effects thereof on male reproductive functions. Well-known and recent advances in methods and techniques used for the assessment of OS will also be highlighted. We will furthermore consider the current evidence regarding the association between OS and ejaculatory abstinence period, as well as the potential mechanisms involved in the pathophysiology of various systemic diseases such as obesity, insulin resistance, hypertension, and certain mental health disorders which have been shown to cause OS induced male infertility. Finally, special emphasis will be placed on the potential for transferring and incorporating research findings emanating from different experimental studies into clinical practice.

## Current Approaches and Methods in the Assessment of OS Markers in Sperm and Semen

There are several methods to measure ROS in the laboratory setting. These include: the the indirect measurement of via enzymatic antioxidant levels (CAT), superoxide dismutase (SOD), glutathione peroxidase (GPx), reduced glutathione (GSH) and Thiobarbituric Acid Reactive Substances (TBARS) via by means of spectrophotometric measurement; the direct measurement of total antioxidant capacity (TAC) *via* Mioxys; the use of chemiluminescence to detect ROS species via the membrane permeable reagent, luminol; and the assessment of ROS and reactive nitrogen species (RNS) molecules *via* florescent markers (27) which can be measured *via* using e.g., flow cytometry and fluorescence microscopy.

Seminal plasma is well-endowed with various ROS and thus the TAC of sperm is an example of an indirect method of assessing ROS (28, 29). Antioxidant enzymes such as CAT, SOD, GSH and GPx represent the TAC of sperm (29), and low levels have been discovered in males with impeded fertility (29). Several reagents are used per assay in order to measure the activity of these antioxidant enzymes.

### Superoxide Dismutase

- The SOD enzyme assay is dependent on the spectrophotometric assessment of superoxide (${\text{O}}_{2}^{\;-•}$).
- The assay uses two chemicals, 6 hydroxydopamine (6HD) and diethylenetriaminepentaacetic acid (DETAPAC), to generate ${\text{O}}_{2}^{-•}$ anions, which are reduced in the presence of SOD.
- The reaction yields a colorimetric signal where samples with reduced amounts of SOD emit a lesser signal that can be measured via colorimetry (30).

### Catalase

- CAT is quantified by adding hydrogen peroxide (H_2_O_2_) to the sample and analyzing the rate of decomposition of CAT, which is proportional to a reduction in the absorbance reading generated by the instrument (29, 31).

### Reduced Glutathione

- GSH can be determined by adding 5,5′-dithiobis-2-nitrobenzoic acid (Ellman's reagent) to the sample.
- This reagent reacts with the thiol groups of GSH which convert 2-nitro-5-thiobenzoate (NTB^−^) to NTB^2−^, thereby producing a yellow color, which can subsequently be quantified (29).

### Glutathione Peroxidase

- GPx is measured by adding glutathione reductase (GR) to the sample after which nicotinamide adenine dinucleotide phosphate (NADPH) is transformed to a reduced state, allowing a decrease in absorbance to be subsequently measured (30, 32).

### Lipid Peroxidation

- OS leads to cellular injury via several mechanisms, with lipid peroxidation being a prominent one. Malondialdehyde (MDA) is a typical product of lipid peroxidation. Polyunsaturated fats yield lipid peroxides, which, due to their instability, disintegrate into several compounds including reactive carbonyl compounds, e.g., MDA. In addition, biological specimens undergoing OS contain TBARS such as aldehydes and hydroperoxides, which increase with increasing OS. MDA forms adducts with TBARS, thiobarbituric acid in particular, with which it forms a 1:2 adduct. This MDA-TBA complex, forming under acidic, high temperature (90–100°C) conditions, elicits a colorimetric reaction.
- Additionally, 4-hydroxy-nonenal (HNE) and Isoprostanes (F2-isoprostane, 15-(S)-8-isoprostagladin F2α) are by-products of PUFA, and are also considered as indices of lipid peroxidation (33, 34). These products can be measured via enzyme-linked immunosorbent assay (ELISA), gas chromatography mass spectrometry (GC-MS) or by liquid chromatography- mass spectrometry (LC-MS) (35).

### Total Antioxidant Capacity

- TAC highlights the crucial role of antioxidant enzymes in counterbalancing ROS generation and therefore can be a powerful tool in determining the redox status of a sample (36). However, the measurement of individual antioxidant enzymes can be time consuming and costly. Additionally, it does not generate a direct measure of the total level of ROS in a system (37).

### Oxidative Reduction Potential

- Oxidative reduction potential (ORP) is a direct analysis of OS (38). It measures the transfer of electrons from a reductant to an oxidant (38).
- ORP has been shown to be negatively correlated with sperm concentration and total sperm count (38).
- This analysis does not rely on any biomarker for OS and allows for the quantification of all oxidants and antioxidants in a given sample (38).
- The Male infertility Oxidation System (MiOXSYS) can be used to accurately measure ORP (39).
- The system consists of an electrochemical cell with a counter and a reference electrode, as well as an impedance electrometer (39).
- It measures static ORP (sORP) which is indicative of the existing balance between total oxidants and reductants in a sample, and the antioxidant capacity reserve (cORP). Samples of high sORP levels indicate an imbalance that suggests the presence of OS (39).
- Unlike chemiluminescence (see later) where ROS levels have a significantly short half-life, ORP measurements are stable for up to 120 min.
- Additionally, MiOXSYS sORP's ability to measure all oxidants and reductants makes it clinically meaningful in the diagnosis of cases of male infertility that is associated with high ROS levels (40).

### Chemiluminescence

- Chemiluminescence measures light that is emitted in a reaction when reagents are added to a biological sample (41).
- Membrane permeable probes such as luminol and lucigen react with ROS and generate a luminescent signal. Luminol measures H_2_O_2_, ${\text{O}}_{2}^{-•}$ and other ROS as these oxidants bind to luminol and they become univalently oxidized luminol radicals, or an oxidative event occurs which can be measured by a chemiluminometer.
- The oxidative event is a one-electron reaction which occurs due to the presence of e.g., endogenous H_2_O_2_ peroxidase.
- Unstable radicals are generated which react with oxygen molecules in their ground state, producing ${\text{O}}_{2}^{-•}$. The ${\text{O}}_{2}^{-•}$ additionally oxygenates luminol radical species, which then creates an unstabe endoperoxide that degrades and causes a light emission (41).
- Luminol and Lucigen are two probes that are used in chemiluminescent assays to detect ROS. Lucigenin is best suited to detect ${\text{O}}_{2}^{-•}$ as it is positively charged, which renders it membrane-impermeable and allows it to react with ${\text{O}}_{2}^{-•}$ in the extracellular space (29, 42). Unlike Lucigen, Luminol is uncharged and is therefore membrane permeable. This allows it to react with ROS in both the intra and extracellular spaces (29). Luminol reacts with a variety of ROS including ${\text{O}}_{2}^{-•}$, H_2_O_2_ and hydroxyl radicals (OH^•^) (40). This probe, however, is unable to differentiate between the types of ROS (40).
- Chemiluminescent assays have been used to show a negative association between an increase in ROS levels and sperm parameters. These parameters include sperm motility, viability, morphology and concentration (29). A variety of factors may influence the data generated by the chemiluminescence assays, which include: the presence of leukocytes in the sperm sample, sperm incubation time, the pH, and contamination of the seminal plasma. It should also be noted that both sample and probe concentrations also affect luminescence, thus it is important to have a fixed probe concentration for varying concentrations of sperm.
- Chemiluminescence assays are sensitive, which is ideal as sperm generally produce low concentrations of ROS (40, 43). The results of the assay may be reliable in samples with sperm of a concentration ±1 million/mL, as the sensitivity of the assay declines significantly at greater concentrations (44).

### Flow Cytometry

- Flow cytometry involves the use of florescent markers to measure ROS and RNS (45, 46). Contradictory to chemiluminescence, florescent techniques have a higher accuracy, specificity and reproducibility rate.
- Flow cytometry allows for the exclusive focus on spermatozoa (46). Additionally, a large number of cells can be analyzed at once.
- However, the utilization of florescent probes requires expensive equipment. The data generated does not quantify ROS but rather is indicative of the percentage of cells displaying a high ROS activity.
- A florescent probe like 2,7-dichlorofluorescein diacetate (H_2_DCFDA) penetrates the spermatozoa and indicates H_2_O_2_ concentrations, as H_2_O_2_ de-esterifies in the presence of DCFH and forms fluorescent 2,7-dichlorofluorescein (DCF) (47). DCF fluoresces and this can be measured.
- Dihydroethidium/hydroethidine (DHE) is a non-florescent probe that is oxidized by a variety of reactive oxygen and nitrogen species. This probe is primarily used to visualize ${\text{O}}_{2}^{-•}$ production. The probe allows for hydroxylation which generates a red florescent emission that, in turn, stains the mitochondrial and nuclear DNA of sperm. Utilizing DHE is advantageous as it allows for a clearer insight into the frontier role of the mitochondria in ROS production (48).

## Origins of ROS in Semen

Leukocytes often exist throughout the male genital tract and the presence of small quantities in the ejaculate represents a normal finding. However, when the level of the peroxidase-positive leukocytes goes beyond 1 × 10^6^/mL, leukocytospermia is present (3). Polymorphonuclear neutrophils, the most common type of seminal leukocytes, represent a primary endogenous source of ROS in semen. Stimulation of these leukocytes during an accessory gland or genitourinary infections triggers a prominent increase in oxygen consumption and ROS generation (49, 50), which impose OS on the engulfed pathogens as part of the defense mechanisms. In patients with genital tract infection, the presence of elevated levels of leukocytes in semen has been associated with suboptimal semen quality and impaired fertility potential (51–54). Several hypotheses have been suggested to elucidate the role of leukocytospermia in enhancing the generation of ROS by human spermatozoa, but the precise molecular mechanism remains unclear. However, the direct sperm-leukocyte interaction, as well as the inflammatory mediators released by infiltrating leukocytes and bacteria have been proposed to be implicated in altering the aerobic metabolism of spermatozoa (55, 56). Furthermore, the presence of bacteria in semen has been suggested to attenuate the ROS scavenging capacity of spermatozoa. Such effect may vary widely depending on the virulence of the pathogen as well as the sperm subpopulation (56, 57).

Additionally, spermatozoa with excess residual cytoplasm retaining along the midpiece are considered immature and functionally defected cells. Cytoplasmic residues contain an increased amount of enzymes particularly glucose-6-phosphate dehydrogenase (G6PD) and NADPH oxidase (25). The excessive presence of G6PD results in the generation of a significant amounts of NADPH, which represents a primary source of electrons essential for the reduction of molecular oxygen to ${\text{O}}_{2}^{-•}$ (50).

Mature spermatozoa themselves have also been suggested to play a critical role in the production of ROS, probably as an end-product of cellular metabolism (26). Mitochondria play a key role in the synthesis of ROS via the activation of the electron transport mechanism at the inner surface of the mitochondrial membrane during oxidative phosphorylation process. This crucial cellular process involves the transfer of electrons and the reduction of oxygen molecules, producing ${\text{O}}_{2}^{-•}$ (58). Furthermore, mitochondrial membranes are highly rich in PUFAs which are primary substrates for ROS attack and lipid peroxidation. This concurrently triggers the production of highly reactive lipid aldehydes which covalently interact with electron transport chain proteins, initiating vicious cycles of increased rates of mitochondrial ROS generation (59).

Another source of OS is a varicocele testis. Varicocele is an abnormal distention of the testicular veins in the pampiniform plexus within the spermatic cord. Clinical varicocele is a major contributor to male infertility, affecting as many as 40% of men with primary infertility and up to 80% of men with secondary infertility (60–62). There is considerable evidence that suggest the implication of OS in the underlying mechanism of infertility in varicocele patients. Numerous studies have reported significantly increased levels of ROS in the semen of varicocele patients (63–65). Varicocele patients have further shown considerably elevated levels of 8-hydroxy-2′- deoxyguanosine, which is a sensitive biomarker of oxidative DNA damage (66). These findings are supported by the recent evidence highlighting the effectiveness of varicocelectomy in improving sperm motility, DNA integrity, antioxidant capacity and pregnancy rates (67–70) in infertile men with varicocele.

Besides the above-mentioned endogenous sources of ROS, a growing list of potential lifestyles and environmental factors have been suggested to contribute to the extreme generation of ROS in semen. These include cigarette smoking (71), alcohol consumption (72) and electromagnetic radiations (73).

## Physiological Roles of ROS

**Figure 1:**
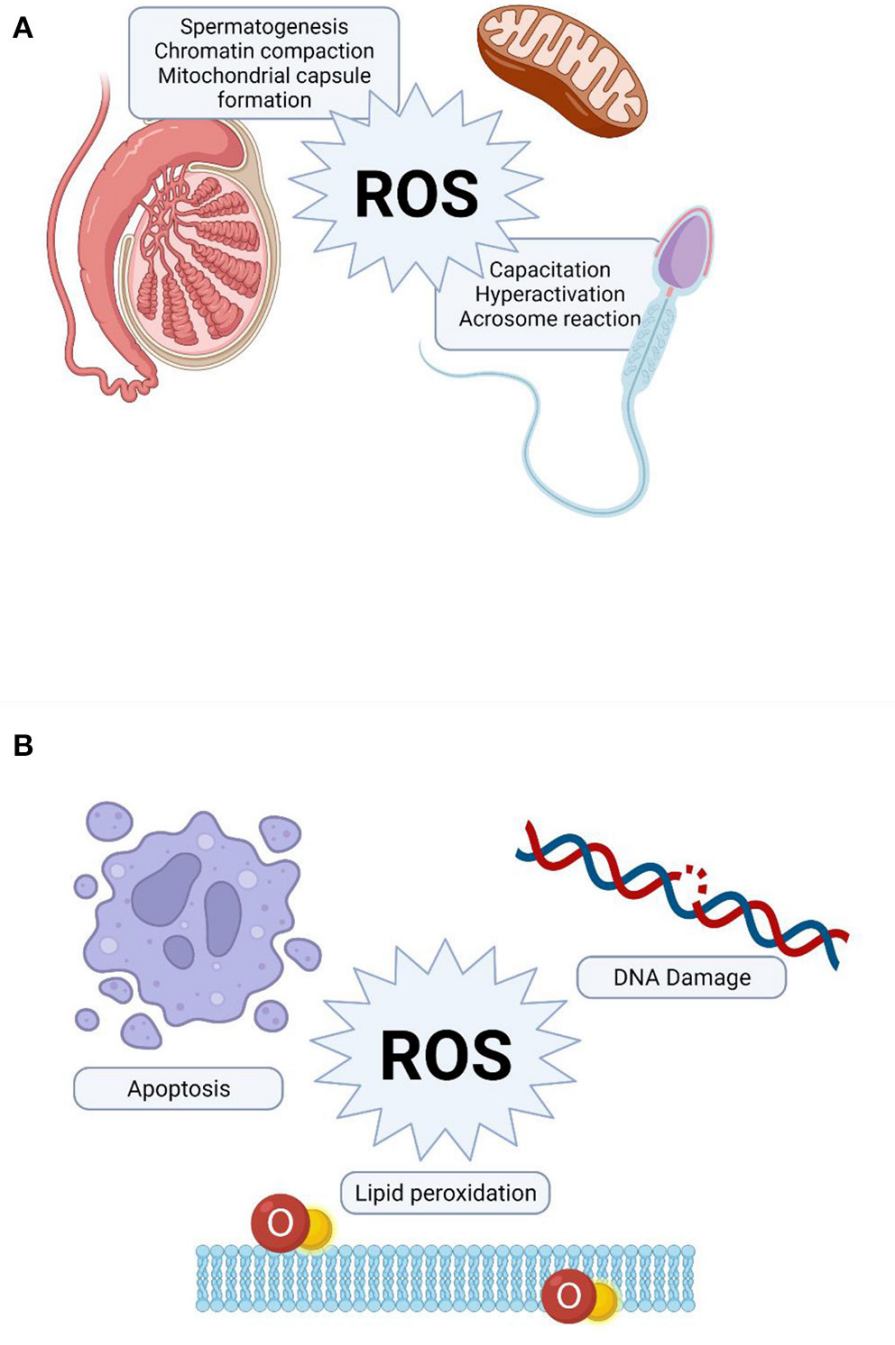
Role of reactive oxygen species (ROS) in sperm function. **(A)** Physiological role of ROS, **(B)** Pathological effect of ROS on sperm function.

During spermatogenesis and epididymal transit, appropriate amounts of ROS are required for the oxidation of cystein-thiol groups essential for chromatin compaction and stabilization in spermatozoa (25). As shown in Figure 1A, ROS are furthermore implicated in the production of the mitochondrial capsule through the oxidation of protein thiols catalyzed by phospholipid hydroperoxide glutathione peroxidise. This enzyme is finally converted to a cross-linked structural protein, comprising a significant component of the mitochondrial sheath of mature spermatozoa (26, 74).

Hyperactivation is a sperm motility pattern characterized by an extremely vigorous whiplash-like flagellar beating, which enhances the ability of the sperm to penetrate the cumulus cell layer surrounding the oocyte (75, 76). The exact molecular mechanism underlying sperm hyperactivation has not been fully elucidated. However, a mild, constant generation of ${\text{O}}_{2}^{-•}$ has been shown to be crucial not only for the initiation, but also the preservation of the hyperactivated state (75). Furthermore, a significant increase in the proportion of spermatozoa displaying hyperactivated motility was observed when the incubation medium had been supplemented with an optimum concentration of H_2_O_2_ (77).

Capacitation is a crucial physiological process of sperm maturation occurs during its passage through the female reproductive tract to enable sperm-oocyte interaction (78). During this process, ROS act as essential intracellular messengers to activate downstream signaling molecules mediating significant changes in the membrane architecture (i.e., membrane destabilization, permeability and lipid redistribution), which are prerequisite for successful fertilization (79). Furthermore, the established role of ROS in modulating tyrosine phosphorylation during capacitation and other relevant processes is tightly regulated by complex cross-talks between various signaling pathways, including the cAMP/PKA and the extracellular signal-regulated kinase pathways (79–81).

In contrast to sperm capacitation, acrosome reaction is an irreversible process which involves the release of acrosomal hydrolytic enzymes to attain sperm oocyte fusion (82). *In vivo*, the sperm-zona pellucida binding triggers a cascade of signal transduction reactions that culminates in an significant increase in intracellular Ca^2+^ levels required for acrosomal exocytosis (83). The generation of both ${\text{O}}_{2}^{-•}$ and H_2_O_2_ within physiological limits is essential to elicit acrosome reaction through the activation of tyrosine phosphorylation of potential target proteins particularly on the pre-equatorial region of the sperm head surface (84). The biochemical cascade of events involved in triggering the acrosome reaction appears to share some underlying features with those identified for capacitation, such as Ca^2+^ influx and the activation of adenylate cyclase and cAMP as well as the phosphorylation of reasonably similar target substrates (25, 81, 85).

## Pathological Effects of ROS on Spermatozoa

Remarkably increased ROS levels have been detected in 25–40% of semen samples of infertile patients (28, 86, 87). The extent of oxidative damage to spermatozoa may vary significantly among infertile men depending on the concentrations and properties of reactive molecules, length of exposure, antioxidant efficiency as well as surrounding temperature and oxygen tension. High concentrations and prolonged exposure to ROS causes extensive damage to various integral cellular biomolecules (Figure 1B), including proteins, lipids and nucleic acids, which ultimately hampers multiple cellular functions (25, 26, 49, 88).

Sperm plasma membrane contains larger amounts of PUFAs, particularly docosahexaenoic acids, which are required to maintain optimal fluidity essential for multiple membrane fusion events. However, theses fatty acids act as potential substrates for peroxidation as they comprise methylene groups with highly reactive hydrogen atoms, thereby enhancing the susceptibility of sperm to oxidative damage (89). Lipid peroxidation is a chain reaction initiated when ROS, particularly OH^•^ and Hydroperoxyl (HO_2_) generated from ${\text{O}}_{2}^{-•}$, combine with a hydrogen atom from a fatty acid to produce a lipid radical. These unstable radicals react rapidly with oxygen molecules to form peroxy fatty acid radicals, which are thereafter converted into lipid peroxides. In the presence of a transitional metal ion, lipid peroxide is catalyzed into OH^•^ (90), which have the ability to attract electrons from other PUFAs to generate new radicals and thereby propagating the lipid peroxidation chain reaction (25). This chain reaction is halted when the radicals react with each other to create a non-reactive product called MDA. This by-product is widely used as a biomarker to estimate the extent of peroxidation damage to spermatozoa (91). Another product of interest in the assessment of lipid peroxidation in any biological sample are the Isoprostanes (IsoPs).

The sustained lipid peroxidation chain reaction results in progressive loss of membrane fatty acids, with consequent decrease in membrane fluidity, increased non-specific permeability to ions, and inhibition of membrane bound receptors and enzymes (23).

Besides their role in mediating lipid peroxidation, ROS especially nitric oxide (NO^•^), ${\text{O}}_{2}^{-•}$ and OH^•^ (92), have also been shown to attach to sperm DNA molecules causing excessive modifications and deletions in their nucleotide bases and strand breakages, along with other multiple genotoxic effects (93). Sperm DNA is especially prone to oxidative damage owing to its poor chromatin compaction and incomplete protamination (55, 94). The role of ROS in sperm DNA damaging has further been supported by the significant and positive correlations observed between the levels seminal ROS and the proportions of spermatozoa with fragmented DNA (95, 96).

ROS has also been implicated as an apoptotic stimulus that triggers the mitochondria to release some signaling molecules crucial for the activation of programmed cell death (97). Mature spermatozoa from ROS-positive infertile patients showed substantially elevated levels of apoptosis compared with the control group (98). However, antioxidant therapy has recently shown to reduce the apoptotic response to OS (99).

## OS-Induced Changes in Conventional Semen Parameters

Several studies have been undertaken correlating various OS markers with fertility status (100–102) and also basic sperm quality parameters including morphology (103, 104) and motility (24, 105, 106). In this regard, the SURRG has published the most recent and comprehensive study aiming at establishing statistical correlations between conventional semen parameters obtained with Computer-aided sperm analysis (CASA) and a set of OS and membrane lipid peroxidation variables. The insight gained from this study complement those of previous investigations and contribute additional evidence with respect to the significance of detailed CASA motility, velocity and kinematic parameters in bridging the gap between conventional semen analysis and the oxidative status (107). Results obtained from this study indicates that rapid progressive motility should prove to be particularly valuable sensitive indicator of lipid peroxidation that could be impaired prior to any detectable deterioration in other sperm motion characteristics. In addition, the observed inverse relationship between intracellular sperm ${\text{O}}_{2}^{-•}$ levels and average-path velocity (VAP) is important in furthering our understanding of the possible role of free radicals in constraining the actual rate of sperm forward movement within the female reproductive tract, which is vital for successful fertilization. Similarly, the strong positive correlations between SOD activity and both curvilinear velocity (VCL) and amplitude of lateral head displacement (ALH) established a quantitative framework for detecting the role of OS in the development of spontaneous and premature hyperactivated motility of spermatozoa in the ejaculate.

Continuing along these lines, it is recommended for future studies the predicting of OS markers from conventional basic parameters through the building of linear regression models assist in indicating the extent to which changes in each individual measurement of basic semen analysis are related to changes in the oxidative status. Indeed, OS markers provide valuable clinical insight into vital aspects of sperm functions. However, the prediction of these markers from the core basic parameters could possibly assist in limiting the necessity for these assays, which are complex, highly expensive and lack universal standardization. This would also enhance the applicability of basic semen analysis, which remains the bedrock of any semen diagnosis, as a more cost-effective and efficient approach for the diagnosis of idiopathic and unexplained male infertility.

## Alterations in OS Status During Different Sexual Abstinence Periods

According to the most recent WHO manual for processing and examining human semen (108) and guidelines for the same by other associations such as Nordic Association for Andrology (NAFA) and the European Society of Human Reproduction and Embryology (ESHRE) (109), subjects must remain abstinent for a minimum period of 2 days, but not longer than seven days before collecting a sample for a standard semen analysis. While the NAFA and ESHRE further added that abstinence intervals of 3–4 days is preferred for analyzing human semen. These variations in ejaculatory abstinence periods suggested by different regulatory bodies has instigated a growing concern as to what the precise period of ejaculatory abstinence ought to be for an optimal semen sample. This has prompted several studies to examine the influence of abstinence periods on various semen quality parameters; however, the results are not conclusive.

Given the gap of evidence, members of our SURRG recently published a comprehensive systematic review of literature assessing the relationship between sexual abstinence and a range of semen/sperm quality parameters (110). This review included a total of 30 studies published between January 1979 and December 2016, where the periods of sexual abstinence were categorized into ≤ 1 day, 2–3 days, 4–5 days, 6–7 days and >7 days. Ayad et al. displayed that three studies had considered intracellular ROS concentration as a quality parameter of sperm when evaluating the effect of the abstinence period (38, 111, 112), whereas only one study investigated the relationship with respect to seminal ROS levels (113). These studies collectively showed a general trend of decrease in ROS levels after short abstinence compared with long abstinence. Additionally, only one study (114) had evaluated the relationship in terms of seminal plasma antioxidants and sperm lipid peroxidation. Marshburn et al. reported a considerable improvement in the TAC of seminal plasma after 1 day of sexual abstinence compared to 4 days, whereas lipid peroxidation of the sperm membrane remained unchanged. Furthermore, a study conducted in our laboratory included a hundred normospermic individuals revealed that only 4 h of sexual abstinence could significant increase seminal plasma SOD activity, but did not change the CAT activity or TBARS levels (115). More recently, shortening the abstinence duration from >4 to ≤ 2 days resulted in a significant reduction in the levels of ROS in semen samples collected from 90 patients with unexplained male factor infertility (116). Similar findings were also shown by Okada et al. (117), who collected semen samples from 50 normospermic men each abstaining sequentially for 1 and 4 days. They reported a significant decline in seminal plasma TBARS levels and intracellular oxidative activity after only 1 day of sexual abstinence (117).

**Figure 2:**
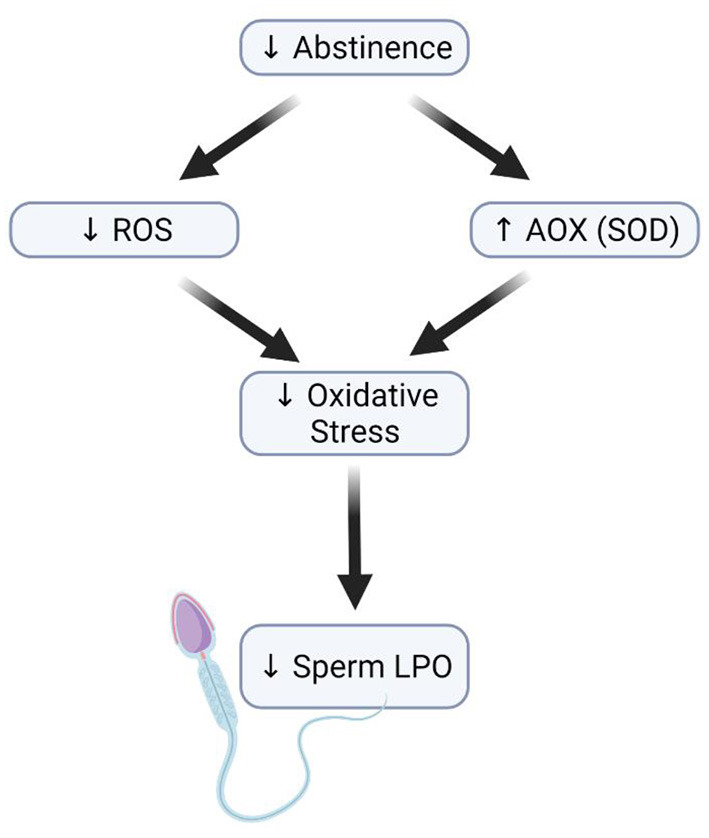
Effect of short abstinence on sperm function. Decrease in the length of abstinence (short abstinence) leads to a reduction in ROS levels and an increased antioxidant level, which consequently reduces the rate of sperm membrane lipid peroxidation.

Despite the limited data available, the weight of evidence indicates that decreasing the ejaculatory abstinence period may minimize the adverse effects of OS on sperm quality and function. In addition to their limited intracellular antioxidant capacity spermatozoa, during their epididymis maturation and storage, are constantly exposed to ROS damage by lipid peroxidation through their PUFA rich plasma membranes. Accordingly, the release of spermatozoa through more frequent ejaculations might be a potential strategy to attenuate the detrimental effects of ROS and improve perm quality (Figure 2) (115, 118).

## Hypertension-Induced OS in Male Infertility

Hypertension, also referred to as elevated blood pressure is estimated to affect about 1.28 billion adults aged 30–79 globally (119), indicating that 16.4% of the world's population is hypertensive. Studies have associated hypertension to some aspects of sperm function (120–123). A group from Brazil that utilized a rat model for renovascular hypertension to investigate the link between hypertension and male infertility observed a decreased sexual behavior and impaired spermatogenesis, which was attributed to imbalances in prolactin, testosterone and follicle-stimulating hormone (FSH) levels (122). Additionally, a group from Italy found elevated levels of clusterin, a glycoprotein linked with abnormal sperm morphology, in a cohort of hypertensive men when compared to normotensive men (123). In the United States, Guo et al. investigated the association between hypertension and semen quality (121). They reported that men with hypertension have one or more semen abnormalities (reduced semen volume (2.1 vs. 3.0 mL, *p* < 0.001), sperm motility (41.0 vs. 47.0%, *p* = 0.008), total sperm count (103.8 vs. 147.0, *p* = 0.005) and total motile sperm count (43.1 vs. 65.9, *p* = 0.003) compared to normotensive men.

Kasman et al. investigated whether the association between male infertility and occurrence of cardiometabolic disease (diabetes, hypertension, hyperlipidemia, and heart disease) is modified by socioeconomics, race, or geographic region (16). They reported that infertile men had a higher risk of incident for hypertension, diabetes, hyperlipidemia, and heart disease regardless of race, region or socioeconomic status. The prevalence and effects of medical comorbidities (hypertension, hyperlipidemia, hyperuricemia and skin disease) on spermatogenesis was determined by Shiraishi and Matsuyama (124) in a group of fertile and infertile men. The prevalence of comorbidities was significantly higher in the infertile men (21.7%) than in the fertile men (9.1%), particularly for hypertension (17.8%), hyperlipidemia (5.9%), hyperuricemia (5.2%), and skin disease (3.0%). Among the infertile men, the reproductive functions were abnormal in the men with comorbidity compared with those without comorbidity. The authors concluded that medical comorbidities are associated with impaired sperm production and suggested that male infertility evaluation offers not only specific therapy to improve semen parameters but also treatment for non-specific medical comorbidities, which may benefit general health status and spermatogenesis restoration. Several other population-based studies have shown the association between male infertility and hypertension (125–127). Animal studies have also thrived in this aspect of reproductive research and have provided evidence that hypertension is associated to male infertility (128–130). Akinyemi et al. reported a significant decrease in serum total testosterone and reduced sperm progressive motility in hypertensive rats. They showed increased OS status in the testes and epididymides of hypertensive rats as evidenced by a significant reduction in total and non-protein thiol levels, glutathione S-transferase (GST) activity with a concomitant increase in DFCH oxidation and TBARS production. Likewise, a decreased testicular and epididymal NO^•^ level with simultaneous elevation in arginase activity was observed in hypertensive rats (128). One of the studies performed in the SURRG laboratory also investigated the association between obesity-induced hypertension and male infertility. Obese hypertensive rats presented with significantly increased levels of serum inflammatory cytokine including, IL-1β, IL-6, IL-12, IL-18 and TNF-α, when compared to the lean group. Also observed were histopathological testicular changes, as there were significant reductions in the seminiferous tubule area (97,807 ± 1,488 μm^2^ vs. 118,347 ± 6,073 μm^2^, *p* < 0.05), seminiferous tubule diameter (354.0 ± 3.0 μm vs. 386.2 ± 9.5 μm, *p* < 0.05), lumen area (19,891 ± 1,717 μm^2^ vs. 30,058 ± 3,639 μm^2^, *p* < 0.01), and lumen diameter (157.8 ± 7.3 μm vs. 191.2 ± 12.2 μm, *p* < 0.05) in the obese hypertensive group compared to the lean group. The obese hypertensive rats also presented with a significantly reduced testosterone: estradiol ratio (130). Another study performed with a hypertensive rat model reported altered sperm quality (sperm motility, normal morphology, sperm count) in hypertensive rats (129). Their findings also showed a reduction in 3β and 17β-hydroxysteroid hydrogenase (3β-HSD and 17β -HSD) activities, as well as testosterone, luteinizing hormone (LH), and FSH levels. An increased ROS and MDA levels were observed with a subsequent decrease in thiol levels, CAT, and glutathione-S-transferase activities (129).

**Figure 3:**
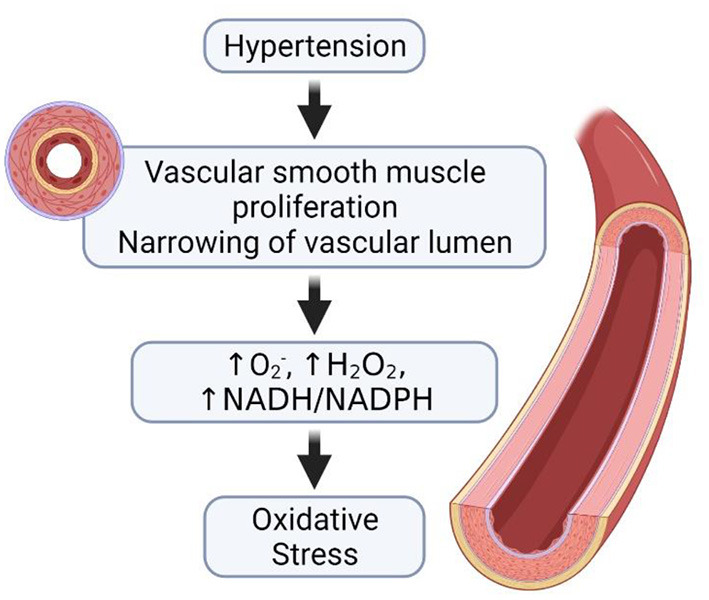
Hypertension and the development of oxidative stress. In the occurrence of hypertension, there is proliferation of the vascular smooth muscles and the narrowing of vascular lumen. The narrowing of the vascular lumen leads to increase in the generation of ROS, thus causing oxidative stress.

Deducing from the above findings, it can be suggested that hypertension impairs male fertility through (i) a reduction in blood flow to the testis, as the oxygen-dependent organ functions in a state of near anoxia, such a decrease in blood flow may very likely have profound effects on the tissue morphology that ultimately would predispose to various forms of hypo-spermatogenesis with consequent compromise in reproductive capability, (ii) altered hormone levels, as the obstructed spermatogenic cells are unable to produce adequate hormone for normal spermatogenesis, (iii) increase in the formation of ROS and a subsequent decrease in antioxidant activities, which consequently lead to OS (Figure 3), and (iv) alteration in the expression of important glycoproteins necessary for normal testicular and sperm morphology.

## Obesity-Induced OS in Male Infertility

Obesity ensues when there is an energy imbalance between the energy consumed and energy expended, therein leading to excessive accumulation of fat (131). Obesity is a multifactorial disorder influenced by genetic or environmental factors, and the incidence has tripled since 1975 as reported by the WHO (131). In 2016, more than 1.9 billion adults of ≥18 years old were overweight, of this, 650 million were obese, indicating that 39% of adults of the world's population aged ≥18 were overweight (39% of men and 40% of women) and 13% were obese (11% of men and 15% of women) (131).

**Figure 4:**
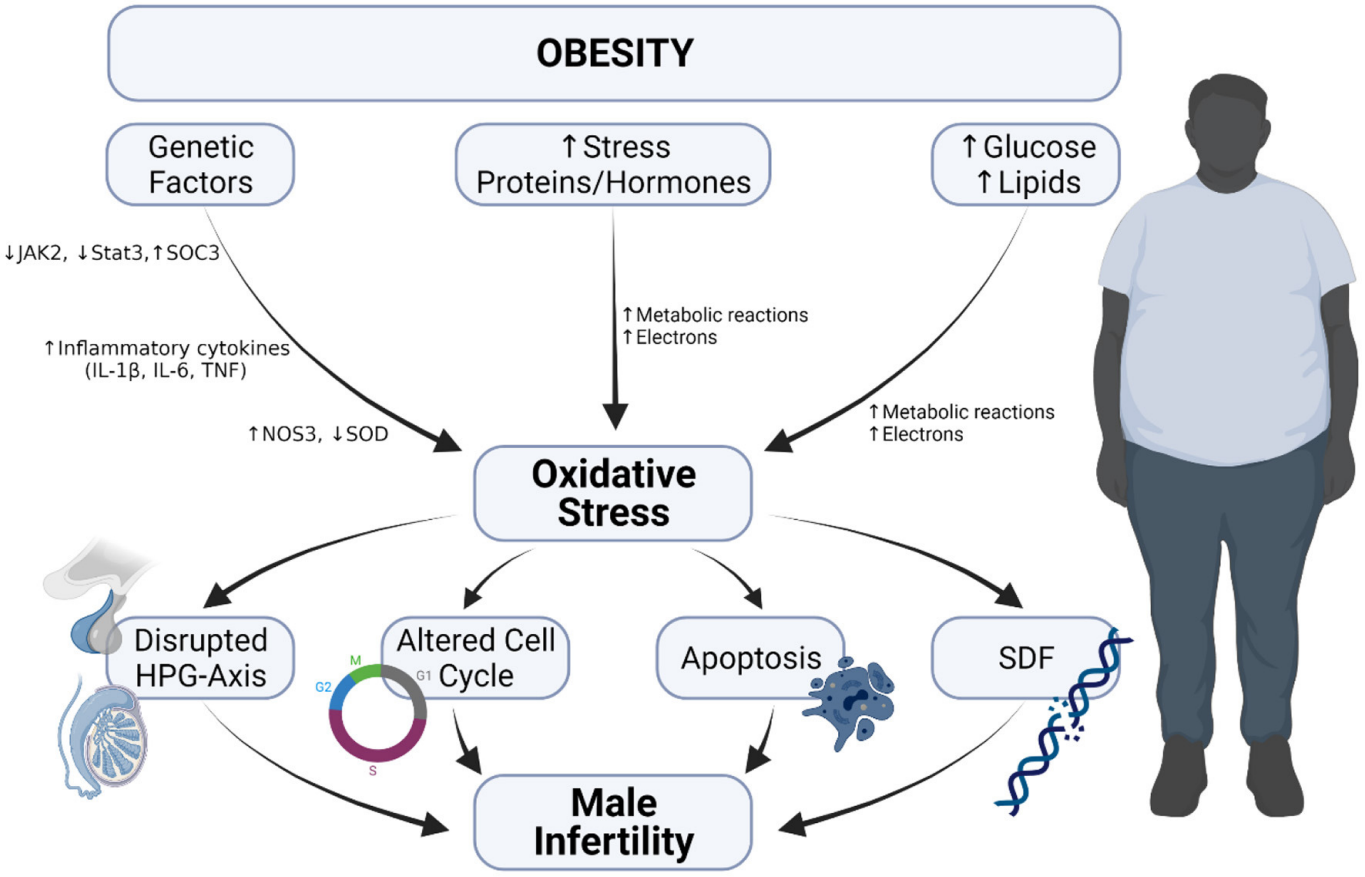
Obesity-induced oxidative stress and male infertility. Obesity-induced oxidative occurs because of (i) increase in metabolic reaction due to elevated glucose and lipids levels, (ii) increase in the level of stress hormones and proteins and (iii) alteration in genetic composition. The occurrence of oxidative stress leads to disrupted hypothalamo-pituitary gonadal axis, induction of apoptosis, increased sperm DNA fragmentation and alters cell cycle, which cumulatively result in male infertility.

The consequence of obesity is not limited to the risk of developing cardiovascular diseases but also include the possibility of male fertility impairment (Figure 4). One of the review articles published by members of our lab explicitly explained how obesity has become a modern man's fertility nemesis (17), as it contributes to reduced semen quality, modified sperm proteomes, erectile dysfunction as well-cause other physical problems (17, 18).

Both animal and human studies have provided evidence that obesity indeed can impair male fertility (15, 132–136). One of the studies performed in the SURRG laboratory assessed the effect of long-term obesity on male reproductive functions (137). After inducing obesity in male Wistar rats for 54 and 60 weeks, respectively, sperm parameters and estradiol levels were adversely altered and some molecular modifications were observed. It was suggested that the instigator of the molecular and proteomic modifications includes an increase in the production of ROS, elevated stress proteins levels and higher levels of redox and inflammatory proteins. Further proteomic analysis of the epididymis and sperm revealed that proteins essential in metabolism, ROS production, stress, inflammation and in the regulation of reproductive function were adversely affected. This means that long term obesity may impair male fertility potential. This is in line with the findings of many others (132, 133). Another study showed that after feeding mice with a high-fat diet (HFD) for 8 weeks, there were manifestations of spermatogenic impairments such as decreased relative testicular weight, impaired testes morphology, and increased percentage of germ cell-depleted tubules. Sperm parameters and functions were also altered (sperm count, sperm motility, sperm viability, reduced serum testosterone), which consequently led to a decrease in pregnancy rate (132). The adverse effects seen were attributed to the occurrence of apoptosis, as there was an increase in caspase 3 activity and a decrease in Bcl-2 activity. Also observed in the obese mice were increased mRNA levels of Xbp-1, Grp78 and CHOP, as these are indicators of endoplasmic reticulum stress, and are believed to be activated by obesity. Abbasihormozi et al. investigated the OS status in the semen samples of men with obesity and type 2 diabetes to validate whether OS in these diseases can influence fertility potential (15). The sperm motility, concentration, total sperm count, and normal sperm morphology were significantly reduced in obese men. The seminal plasma TAC was significantly reduced in the obese group with higher ROS levels, early apoptotic spermatozoa, and increased percentage of sperm with fragmented DNA. Also observed were decreased serum testosterone concentration and increased cortisol levels in the obese group. It was therefore concluded that increase ROS levels and elevated DNA fragmentation in men affected with obesity could be considered as prognostic factors in sub-fertile patients (15). The findings of the study of Abbasihormozi et al. is supported by Raad et al. who also reported impaired sperm quality of men with obesity (135).

Deshpande et al. investigated the effect of both diet-induced and genetically inherited obesity on male fertility in adult male rats. It was reported that the difference between HFD-induced obesity and genetically inherited obesity is shown in the expression of fertility-related hormones and spermatogenesis (138). The authors further explored the expression of genes related to reproductive hormone receptors, leptin signaling molecular biomarkers, pro-inflammatory cytokines, OS and cell cycle mediators in the testis (133). Their findings showed that both types of obesity have altered expression of hormone receptors, cytokines and biomarkers of OS, as well as cell cycle mediators; but the changes are different (133). The differences were seen in the metabolic pathways, and the changes are attributed to the variation in white adipose tissue accumulation (138).

Several other studies, both animal and human have highlighted that in obesity, there is excessive production of ROS, leading to OS and consequently induce apoptosis, which may further lead to reduced reproductive potential or subfertility (139–143). Some showed the disruption of the blood-testis barrier (141) in obesity, while some suggested that the duration have an unfavorable impact on male fertility (142, 143).

## Diabetes Mellitus-Induced OS in Male Infertility

Hyperglycemia can occur because of the inability of the pancreatic beta cells to secrete insulin or as may arise from insulin resistance and variable degrees of inadequate insulin secretion resulting in diabetes and related comorbidities. Given that the global burden of diabetes is continuously increasing, it is estimated that the number of men in their reproductive age that will be affected with diabetes is likewise on the rise, as there is an elevation in the number of childhood and adolescent males with diabetes (144, 145).

**Figure 5:**
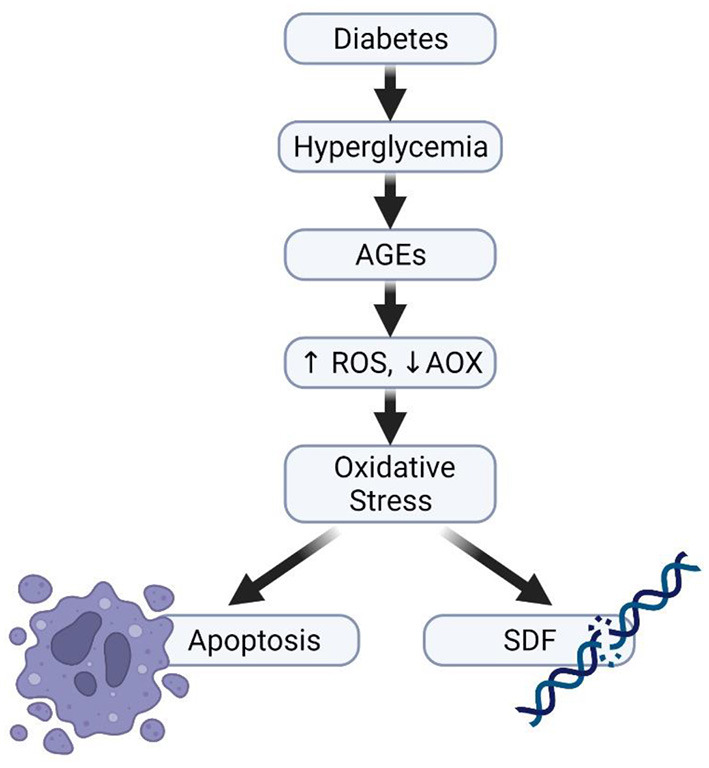
Diabetes and impaired sperm function. Hyperglycaemia can increase the production of advanced glycation end products, thereafter causing imbalance in the ratio of ROS generation and its elimination by antioxidants, thereby resulting in the development of oxidative stress.

Studies have shown that diabetes, both type I and type II induce subtle molecular changes that are essential for sperm quality and function. Bhattacharya et al. reported a significant decrease in sperm motility, including the number of rapid progressive spermatozoa (146). Another study revealed a significant reduction in sperm kinetic parameters and a decrease in the percentage of normal sperm morphology of male diabetic partners (147). Several other studies have also revealed a significant decrease in semen volume, sperm motility and morphology in the semen of diabetic men (148, 149) while some animal studies have equally reported alteration in sperm parameters (150–152). All these effects can partly be explained through the impact of OS, caused by the inequality between ROS production and antioxidant defense mechanisms (Figure 5) (19, 153). The process of how OS leads to male subfertility or infertility has been discussed in detail in the earlier section.

## Psychological Disorders and OS in Male Infertility

The interaction between OS, male infertility and psychological disorders is interesting, as both fertility and the neural system are individually and collectively affected by OS. As mentioned, OS has been implicated as a significant contributor to male infertility (154). OS has independently been implicated in the pathogenesis of various disorders of the brain, such as anxiety disorders, depression, bipolar disorder and schizophrenia (155–157). The contributing factor toward these pathologies is the presence of PUFAs within both the brain and spermatozoa, which are highly susceptible to damage by ROS. In the brain, its lipid-rich composition and extensive oxygen utilization (155–157), amongst other factors, allow for significant susceptibility of the brain to OS-induced damage. The high rates of oxygen consumption allow for substantial free-radical production (157) as the lipids readily provide substrates for oxidation (158). Other factors which contribute toward neuro-susceptibility to OS include, inadequate antioxidant capacities and the availability of redox-catalytic metals (158, 159), the same as with spermatozoa.

The independent susceptibility of the brain to OS has been shown in numerous studies. OS is involved in the pathogenesis of depression and in multiple human studies with patients suffering from depression (156). Findings include decreased antioxidant activity, DNA damage, amplified lipid peroxidation and increased ROS production (156, 160). An interaction between OS pathways and neurogenesis, the uptake of neurotransmitters and neuroinflammation have additionally been suggested (157). This indicates a connection between OS and depression, as the pathogenesis of this disorder involves neuroinflammation and altered neurotransmitter activity. Defective antioxidant capacities and elevated ROS concentrations have been reported in several studies of schizophrenic patients (157). OS contributes to the pathogenesis of schizophrenia through its effect on neuronal excitability and mitochondrial signaling, factors which impart negative influences on neurons and ultimately promote the development of schizophrenia (161). Similar findings have been implicated in the pathogenesis of bipolar disorder, including the involvement of elevated free radicals such as NO, as well as lipid peroxidation and altered antioxidant levels (157, 162).

Psychological stress and anxiety have been shown in various studies to affect male fertility, as well as in the SURRG laboratory (163). The activation of the hypothalamic–pituitary–adrenal axis during stress and anxiety can affect the release of the Gonadotropin Releasing Hormone (GnRH), which can decrease the production of LH, FSH and therefore the release of testosterone (164). In addition, increased psychological stress has been shown to decrease seminal volume, even in healthy individuals (165). The role of NO^•^ in anxiety has not yet been fully elucidated. However, in a study performed on male mice lacking the gene that encodes for NO^•^ synthase 1, these mice were found to have abnormal anxiety levels compared to their counterparts (166, 167). In addition, NO^•^ levels in seminal plasma were shown to be elevated in individuals who scored higher on the State Anxiety Index questionnaire (168). Psychological disorders such as Alzheimer's disease have been shown to cause oxidative damage to glycolytic enzymes as well as enzymes involved in the tricarboxylic cycle during glucose metabolism, thereby affective ATP biogenesis (169). These changes can cause a cascade of oxidative damage that can systemically make its way to the male reproductive system, thereby affecting fertility.

## Discussion and Conclusion

Scientific evidences within and outside the SURRG over the years have revealed that free radicals are neither exclusively beneficial nor exclusively detrimental to the male reproductive functions. Indeed, Redox equilibrium is important for the maintenance of various vital aspects of sperm functionality. However, an imbalance in the generation and elimination of ROS causes impairment in sperm qualities, attributable to oxidative damage due to their limited antioxidant capacity and cell membrane rich in PUFAs. At pathological level, ROS becomes highly reactive, causing substantial damage to various cellular biomolecules such as nucleic acids, proteins and lipids (41). The subsequent series of adverse events include loss of membrane integrity, mitochondrial dysfunction, impaired sperm motility as well as DNA damage and apoptosis resulting in damage of cellular components and pathogenesis of reproductive diseases and infertility (23).

Thus, information obtained from the assessment of OS status could be of great importance in enhancing clinical decision making. However, OS profiling is predominantly used for research settings and is not yet integrated into routine assessment of male infertility. This is primarily due to the lack of universal standards and norms in addition to the assay complexity and high costs (170–173). There is, therefore, a definite need to develop a robust assay that should be simple, validated and easily performed to allow OS screening in a routine andrology laboratories. Further work is also needed to establish cut-off values for the OS key players with sufficient sensitivity and specificity to be clinically useful in the differentiation between fertile and infertile men.

Interestingly, we have established statistical correlations between conventional semen parameters obtained with CASA and a set of OS and membrane lipid peroxidation variables showing that the rapid progressive motility is a valuable sensitive indicator of lipid peroxidation that could be affected, prior to any detectable deterioration in other sperm motion characteristics. Furthermore, the observed inverse relationship between intracellular sperm ${\text{O}}_{2}^{-•}$ levels and VAP is important in advancing our understanding of the possible role of free radicals in constraining the actual rate of sperm-forward movement within the female reproductive tract, which is vital for successful fertilization. These scientific revelations complement those of previous investigators and contribute, additional evidence, with respect to the significance of detailed CASA motility, velocity and kinematic parameters in cementing the relationship between conventional semen analysis and the oxidative status (107).

On the contrary, physiological or homeostatic level of ROS is involved in the activation of intracellular pathways responsible for spermatozoa maturation, capacitation, hyperactivation, acrosomal reaction, chemotactic processes, formation of the mitochondrial capsule and condensation of sperm DNA as well as fusion with the female gamete to ensure fertilization.

The relationship between infertility and potential mechanisms involved in the pathophysiology of various systemic diseases such as insulin resistance, obesity, hypertension, certain mental disorder as well as prolonged ejaculatory abstinence period have also implicated ROS, thereby causing OS induced male infertility in most of these conditions. Kasman et al. (16) have also reported that infertile men had a higher risk of incident for hypertension, diabetes, hyperlipidemia and heart disease regardless of race, region or socioeconomic status. Using animal studies, we have also reported the association between obesity-induced hypertension and male infertility. Obese-hypertensive rats presented with increased levels of serum inflammatory cytokines when compared with control (130). Our studies on the effects of long-term obesity on male reproductive functions suggested that the instigator of the molecular and proteomic modifications includes: an increase in the production of ROS, elevated stress proteins levels and higher levels of redox and inflammatory proteins. Further proteomic analysis of the epididymis and sperm revealed that proteins essential in metabolism, ROS production, stress, inflammation and in the regulation of reproductive function were adversely affected (137). Studies have shown that diabetes, both type I and type II induce subtle molecular changes that are essential for sperm quality and function, but a follow up study by our team projected that the adverse effects caused by diabetes mellitus can partly be explained through the impact of OS, caused by the inequality between ROS production and antioxidant defense mechanisms (152).

On the other hand, psychological disorders have been associated with increased NO^•^ levels and oxidative damage to glycolytic enzymes as well as enzymes involved in the tricarboxylic cycle during glucose metabolism, thereby affecting ATP biogenesis (169). These changes can cause a cascade of oxidative damage that can systemically make its way to the male reproductive system, thereby causing infertility. Deducing from the above findings, it can be suggested that hypertension, obesity, insulin resistance, certain mental disorder and prolonged ejaculatory abstinence period impairs male fertility through increase in the formation of ROS and a subsequent decrease in antioxidant activities, which consequently lead to OS.

## Author Contributions

BA: designed the outline of the manuscript, writing, and editing. TO: contributed to the study design, writing, and editing. BS: writing and editing. NL, YR, and PO: writing. SD: helped with the study design, supervised the team, and reviewed the final version of the manuscript. All authors contributed to the article and approved the submitted version.

## Funding

This work was supported in part by the Al Jalila Foundation.

## Conflict of Interest

The authors declare that the research was conducted in the absence of any commercial or financial relationships that could be construed as a potential conflict of interest.

## Publisher's Note

All claims expressed in this article are solely those of the authors and do not necessarily represent those of their affiliated organizations, or those of the publisher, the editors and the reviewers. Any product that may be evaluated in this article, or claim that may be made by its manufacturer, is not guaranteed or endorsed by the publisher.
